# Mass COVID-19 patient screening using UvsX and UvsY mediated DNA recombination and high throughput parallel sequencing

**DOI:** 10.1038/s41598-022-08034-1

**Published:** 2022-03-08

**Authors:** Dario Palmieri, Amanda Javorina, Jalal Siddiqui, Anne Gardner, Anthony Fries, Richard R. Chapleau, Clarise Starr, Richard Fishel, Wayne O. Miles

**Affiliations:** 1 Department of Cancer Biology and Genetics, College of Medicine and Comprehensive Cancer Center, The Ohio State University, Columbus, OH 43210 USA; 2 Public Health and Preventive Medicine Department, US Air Force School of Aerospace Medicine, Wright-Patterson Air Force Base, OH 45433 USA

**Keywords:** High-throughput screening, Infection

## Abstract

The Severe Acute Respiratory Syndrome Coronavirus-2 (SARS-CoV-2), also known as 2019 novel coronavirus (2019-nCoV), is a highly infectious RNA virus. A percentage of patients develop coronavirus disease 2019 (COVID-19) after infection, whose symptoms include fever, cough, shortness of breath and fatigue. Acute and life-threatening respiratory symptoms are experienced by 10–20% of symptomatic patients, particularly those with underlying medical conditions. One of the main challenges in the containment of COVID-19 is the identification and isolation of asymptomatic/pre-symptomatic individuals. A number of molecular assays are currently used to detect SARS-CoV-2. Many of them can accurately test hundreds or even thousands of patients every day. However, there are presently no testing platforms that enable more than 10,000 tests per day. Here, we describe the foundation for the REcombinase Mediated BaRcoding and AmplificatioN Diagnostic Tool (REMBRANDT), a high-throughput Next Generation Sequencing-based approach for the simultaneous screening of over 100,000 samples per day. The REMBRANDT protocol includes direct two-barcoded amplification of SARS-CoV-2 and control amplicons using an isothermal reaction, and the downstream library preparation for Illumina sequencing and bioinformatics analysis. This protocol represents a potentially powerful approach for community screening of COVID-19 that may be modified for application to any infectious or non-infectious genome.

## Introduction

COVID-19 (coronavirus disease 2019) is an infectious disease whose etiopathogenic agent is the Severe Acute Respiratory Syndrome Coronavirus 2 (SARS-CoV-2) RNA virus^1–4^. As of January 2022, this viral infection has globally affected more than 296 million individuals and claimed almost 5 million lives, according to the World Health Organization (https://covid19.who.int/). The enormous volume of COVID-19 patients has placed significant strain on healthcare systems around the world and led to the confinement of over half of the world’s population. Accommodating state- and country-wide populations resuming pre-pandemic activities will require serial monitoring of large numbers of residents. To address this screening need, a scalable diagnostic test is required that may be used with widely available and existing equipment and infrastructure. In addition, as the proportion of vaccinated individuals grow, COVID-19 testing will need to be incorporated into panel clinical testing rather than the specialized PCR based assay used effectively during large scale pandemic testing.

Here, we describe, and provide reduction-to-practice data, for a mass COVID-19 screening platform: Recombinase Mediated BaRcoding/AmplificatioN Diagnostic Tool (REMBRANDT). This protocol contains a number of benefits over PCR-based and other parallel LAMP and PCR based approaches that maximize output and sensitivity whilst retaining efficiency. Specifically, REMBRANDT uses recombination and repair enzymes to detect and amplify the viral genomic RNA^5^. After its initial development, it has been demonstrated that RPA is a sensitive and versatile amplification method^6^. RPA reactions only require minimal sample preparation and function on a wide variety of human and animal specimens including blood^7^, swabs^8^ and sputum^9^. The sensitivity and processivity of RPA has allowed the development of protocols for the detection of multiple DNA and RNA pathogens including MERS (Middle East Respiratory Syndrome) virus^10^, HIV-1^11^ and Ebola^7^, with limits of detections frequently lower than 100 copies/sample (reviewed in^6^). Relevant to the present study, the efficacy of multiplexed RPA assays has previously been demonstrated^12–14^. Together, a growing body of evidence indicates that RPA can provide a timely, accurate, specific and potentially multiplexable approach for the development of new diagnostic tools. Nonetheless, since RPA does not require primer-specific annealing temperatures, it allows the use of multiple different barcoded primers in similar conditions. By incorporating dual barcoded primers during this process, the REMBRANDT platform enables the amplification of simultaneously tagged individual samples. This step allows the rapid generation of DNA products directly from SARS-CoV-2 in the sample, bypassing the need for first extracting RNA. Utilizing two independent barcoded primers per well enables a single patient sample within a 96-well plate to be independently marked with a well-specific barcode *and* a plate-specific barcode. Once barcoded, the patient samples from multiple 96-well plates can be pooled and purified, ultimately minimizing reagent usage, time and sample-to-sample variation.

Processing these samples together enables rapid library construction using any one of the barcoded Illumina kits. Utilizing Illumina barcodes allows further sample multiplexing to maximize patient numbers that can be screened using Next Generation (NextGen) sequencing. The smaller numbers of barcodes require less time-consuming computational processing, as trimmed sequences can be quickly divided based on barcode and mapped to the SARS-CoV-2N gene. Importantly, this protocol introduces a unique, synthetic SARS-CoV-2N gene sequence into every 96-well plate. This control has identical primer annealing regions to the SARS-CoV-2 gene but contains 6 engineered base pair substitutions that distinguish it from native sequence, providing an internal quality control for the process and a measure of batch effects. The laboratory and computational framework are designed to maximize SARS-CoV-2 detection efficiency while minimizing reagent usage, processing, and turn-around time.

## Results

### The REMBRANDT platform and in vitro substrate testing.

**Figure 1:**
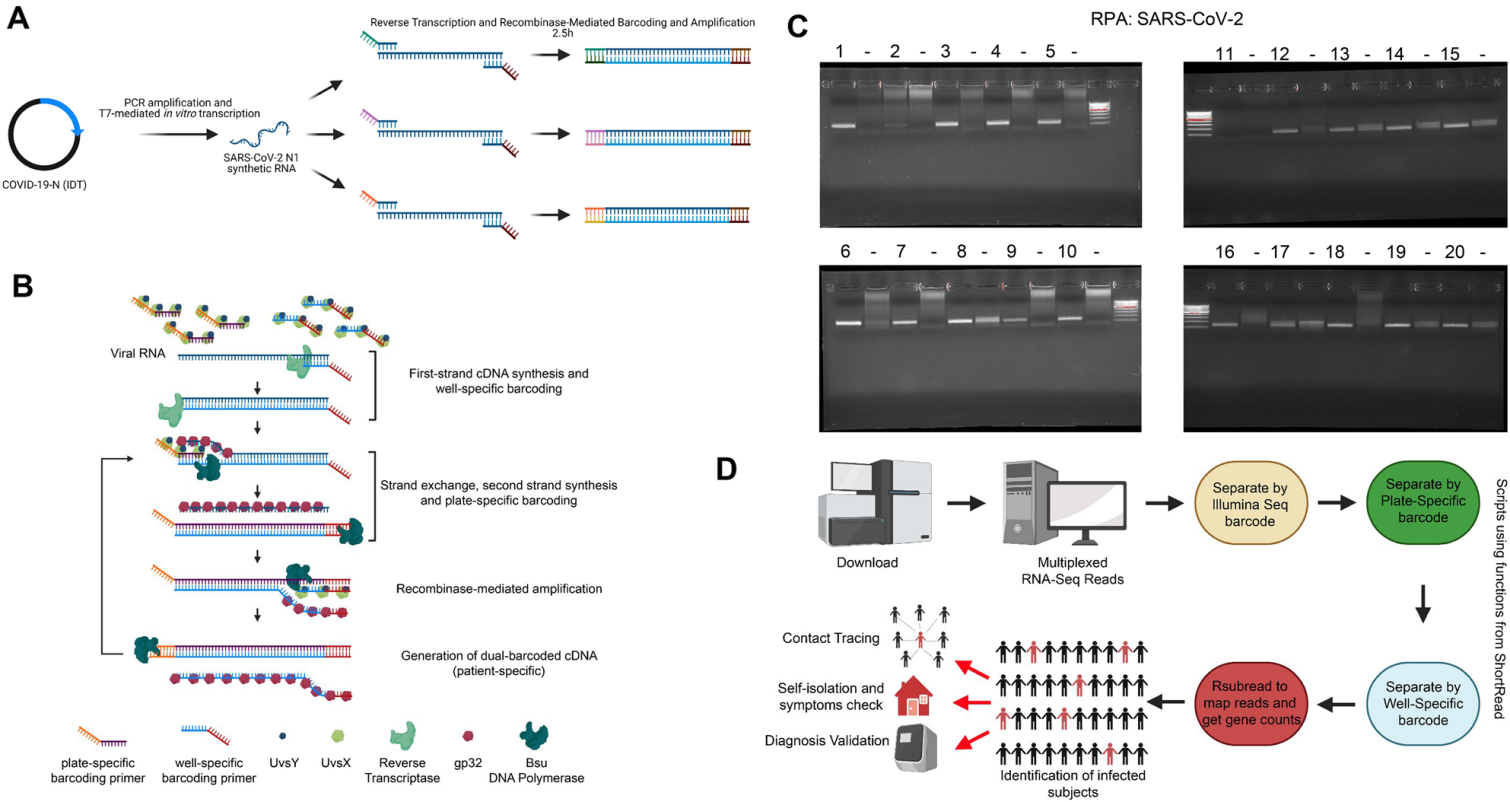
In vitro REMBRANDT testing. (**A**) Generation of in vitro transcribed control and SARS-CoV-2 RNA. Image generated using Biorender (BioRender). (**B**) Schematic detailing the protocol utilized for the isothermal amplification and dual-barcoding of target RNAs. Image generated using Biorender (BioRender). (**C**) Agarose gels using 12 different forward and 8 different reverse barcoded primers for the isothermal amplification of the SARS-CoV-2N gene. Numbered lanes represent unique barcode combinations; – represents water control for each primer pair. (**D**) Schematic detailing the protocol utilized for the bioinformatics analysis of the REMBRANDT RNA-seq data. Image generated using Biorender (BioRender).

To evaluate this strategy for SARS-CoV-2 testing, we first tested whether this approach could identify synthetic SARS-CoV-2 sequences. For these assays, we generated T7-flanked PCR products of the SARS-CoV-2N gene. These were used to make and purify RNA for pilot experiments (Fig. 1A). These RNA fragments were then used in an isothermal amplification reaction using UvsX and UvsY (Fig. 1B) followed by strand invasion and second strand synthesis mediated by GP32 and BSU DNA polymerase (Fig. 1B). This highly efficient reaction catalyzes the rapid synthesis of dual barcoded SARS-CoV-2 regions (Fig. 1B). To test whether this approach works on in vitro generated RNA, we measured the isothermal reaction and DNA production from forward and reverse primers of SARS-CoV-2 that contain different barcodes. As shown in Fig. 1C, each of the primer pairs tested generated a product of the correct size (Fig. 1C: numbered, primer combinations detailed in Table 1); however, the negative control sample for each only produced non-specific bands that did not contain amplified regions. Using this approach, we then generated a computational pipeline that would allow the rapid identification of SARS-CoV-2 positive patients from NextGen sequencing (Fig. 1D).

**Table 1:** Primer combination for in vitro REMBRANDT testing.

Reaction number	Forward Primer	Reverse Primer
1	COVID N FOR, BC 1	COVID N REV, BC 1
2	COVID N FOR, BC 2	COVID N REV, BC 1
3	COVID N FOR, BC 3	COVID N REV, BC 1
4	COVID N FOR, BC 4	COVID N REV, BC 1
5	COVID N FOR, BC 5	COVID N REV, BC 1
6	COVID N FOR, BC 6	COVID N REV, BC 1
7	COVID N FOR, BC 7	COVID N REV, BC 1
8	COVID N FOR, BC 8	COVID N REV, BC 1
9	COVID N FOR, BC 9	COVID N REV, BC 1
10	COVID N FOR, BC 10	COVID N REV, BC 1
11	COVID N FOR, BC 11	COVID N REV, BC 1
12	COVID N FOR, BC 12	COVID N REV, BC 1
13	COVID N FOR, BC 1	COVID N REV, BC 1
14	COVID N FOR, BC 1	COVID N REV, BC 2
15	COVID N FOR, BC 1	COVID N REV, BC 3
16	COVID N FOR, BC 1	COVID N REV, BC 4
17	COVID N FOR, BC 1	COVID N REV, BC 5
18	COVID N FOR, BC 1	COVID N REV, BC 6
19	COVID N FOR, BC 1	COVID N REV, BC 7
20	COVID N FOR, BC 1	COVID N REV, BC 8

## REMBRANDT is able to detect COVID-19 in patient samples

**Figure 2:**
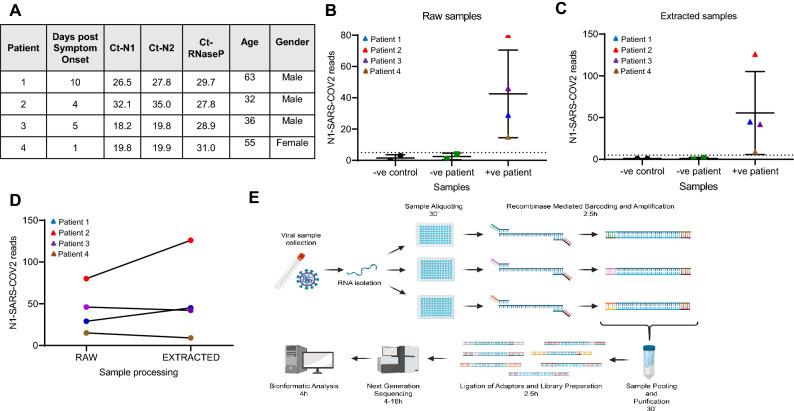
REMBRANDT can identify SARS-CoV-2 positive patient samples from raw and extracted patient samples. (**A**) Patient information from tested individuals including age, gender, days of test following symptoms and COVID and RNaseP Ct values from CDC COVID assay. (**B**) Number of SARS-CoV-2 reads mapped to negative control (-ve control), negative patients (-ve patient samples) and SARS-CoV-2 positive patients (+ve patients) from raw samples. Graph generated using Prism v: 9.2.0 (Home-GraphPad). (**C**) Number of SARS-CoV-2 reads mapped to negative control (-ve control), negative patients (-ve patient samples) and SARS-CoV-2 positive patients (+ve patients) from RNA extracted samples. Graph generated using Prism v: 9.2.0 (Home-GraphPad). (**D**) Comparison of the number of SARS-CoV-2 reads from each patient comparing raw and extracted patient samples. Graph generated using Prism v: 9.2.0 (Home-GraphPad). (**E**) Schematic detailing the REMBRANDT pipeline for testing for COVID-19. Image generated using Biorender (BioRender).

Following these proof-of-principle in vitro-based assays, we next evaluated the capacity of REMBRANDT to identify SARS-CoV-2 in patient samples. These experiments were conducted in a laboratory under conditions consistent with Clinical Laboratory Improvement Amendment (CLIA) certification, to accurately mirror current SARS-CoV-2 screening conditions. For this analysis, we utilized two negative controls (RNAse P and non-templated) and the T7 SARS-CoV-2N RNA as a positive control. To test the efficiency of the REMBRANDT pipeline to detect SARS-CoV-2 positive patients, we profiled 6 patient samples, 2 negative (> 40 Ct value CDC assay) and 4 positive samples (Fig. 2A), from raw (RNA unextracted) and RNA extracted specimens. The SARS-CoV-2 positive patients were selected to represent the spectrum of patients being currently tested. We choose patients with new symptoms (Day 1), existing symptoms (Day 4 and 5), and diminishing symptoms (Day 10). These patients also had wide ranging SARS-CoV-2 Ct values (18.2–35) based on RT-PCR analysis, but little variation in control gene levels (RNaseP, 27.8–31) (Fig. 2A). The clinical diagnosis of each patient was blinded for this analysis.

Following the REMBRANDT reaction, library construction and RNA-sequencing on the Illumina MiSeq, we utilized the REMBRANDT informatics pipeline to separate patient-specific barcodes and mapped the reads to the SARS-CoV-2 amplicon. Using this approach, REMBRANDT can clearly segregate SARS-CoV-2 positive patients from controls in raw (unextracted) (Fig. 2B) and RNA extracted (Fig. 2C) samples (primers detailed in Table 2 and 3). This data enabled us to set a threshold of at least 5 N1-SARS-CoV-2 reads was required to identify positive patients (Fig. 2B and 2C). In our analysis, we found substantial separation of positive patients from both negative controls and SARS-CoV-2 negative patients, in both raw and extracted samples. This approach can discriminate low SARS-CoV-2 positive patients from background, without the need for additional sequencing depth. Interestingly, we find little correlation between SARS-CoV-2 levels from PCR analysis and REMBRANDT reads (Fig. 2A and Fig. 2B/C). We note that Patient 2 and Patient 4, which have the lowest and highest Ct-N1 and Ct-N2 COVID-19 values by PCR, show inverse N1 REMBRANDT reads (Fig. 2A and Fig. 2B/C). We find that patient samples with strong Ct-RNaseP values (Patient 2 and 4, Fig. 2A and Fig. 2B/C), produce clearer SARS-CoV-2 results. This suggested that REMBRANDT may be best utilized as a pipeline for identifying positive patients and not quantifying infection levels. To evaluate the necessity for RNA extraction in the correct identification of patients, we compared SARS-CoV-2 read depth and found little difference between inputs (Fig. 2D). These findings suggest that raw (unextracted) patient samples can be utilized in the REMBRANDT approach, which has processing benefits and overcomes existing reagent bottlenecks.

**Table 2:** Forward primers for REMBRANDT test runs.

Forward primer	Sequence
COVID N FOR, BC 13	TGT AAA ACG GCC AGT TAT CTG TGG ACC CCA AAA TCA GCG AAA TGC ACC CCG
COVID N FOR, BC 14	TGT AAA ACG GCC AGT GCC GAA TGG ACC CCA AAA TCA GCG AAA TGC ACC CCG
COVID N FOR, BC 15	TGT AAA ACG GCC AGT TAC TGC AGG ACC CCA AAA TCA GCG AAA TGC ACC CCG

**Table 3:** Reverse primers for REMBRANDT test runs.

Reverse Primer	Sequence
COVID N REV BC 9	CAG GAA ACA GCT ATG ACC TTA GTG GGC GTT CTC CAT TCT GGT TAC TGC CAG TTG
COVID N REV BC 10	CAG GAA ACA GCT ATG ACC GCA TAG TGC GTT CTC CAT TCT GGT TAC TGC CAG TTG
COVID N REV BC 11	CAG GAA ACA GCT ATG ACG AAG CGA TGC GTT CTC CAT TCT GGT TAC TGC CAG TTG

## Discussion

SARS-CoV-2 is a highly infectious single-stranded RNA virus. However, increasing evidence suggests that the vast majority of infected individuals display few or very mild symptoms^15^. These people may still spread the virus for over 10 days after the initial contagion^16^. Even as the number of people vaccinated against SARS-CoV-2 grows, the rapid identification and quarantining of infected individuals will remain a key measure to contain the spread of SARS-CoV-2. One of the major current limitations of population testing is the availability of reagents. The current clinical standard for COVID-19 diagnosis (qRT-PCR) requires suitable equipment for the amplification of the viral RNA and the detection of the infection. Each of these steps significantly slows sample processing and limits the number of tests that can be performed in a day. For this reason, new diagnostic tools are needed that are efficient and scalable.

To address this need, the scientific community has delivered a remarkable and unprecedented number of assays to diagnose COVID-19^17–19,26^. One particularly promising approach uses viral RNA reverse transcription and patient-specific barcoding of the single strand of cDNA, followed by cDNA amplification and NGS analysis^9^. This approach, although promising, requires 1 barcode per patient rather than multiplexing barcodes. That means to screen 10,000 patients, one requires 10,000 barcodes. Furthermore, these large primers with large barcodes are likely to vary significantly in their amplification efficiency, making the individual testing of these primer sets essential. To overcome these issues, Schmid-Burg and colleagues developed a highly scalable Reverse-Transcription Loop-mediated Isothermal Amplification (RT-LAMP) method for population sequencing^10^. However, this approach relies on the use of 3 proprietary enzymes, whose availability might limit its scalability. In contrast, the 4 key enzymes required for the REMBRANDT pipeline, including UvsX, UvsY, GP32 and BSU DNA polymerase, are widely available from both commercial and academic sources. Diversifying the approach for widespread COVID-19 testing would ease some of the significant pressure on the supply chain for standard PCR based protocols.

REcombinase Mediated BaRcoding and AmplificatioN Diagnostic Tool (REMBRANDT) builds on these principles to double barcode each patient sample using an isothermal RT-RPA reaction. The combinatorial use of multiple forward and reverse barcodes, one per patient and one per plate, enables 192 primers to generate 9,216 patient-specific combinations. This number can then be further multiplexed and amplified with 12 Illumina barcodes utilized during library construction. Based on a 50 million read sequencing run on a MiSeq Illumina platform, ~ 500X coverage for each combination of barcodes is possible in an individual, which is significantly above our reads from positive patients (20–150). As it is unlikely that a single testing facility would have > 100,000 samples to process and analyze each day, necessitating either smaller runs, or the pooling of samples from multiple sites. This straightforward approach does not require specialized equipment for patient detection and library construction. We therefore have confidence that this system could be readily implemented in most communities, including those with limited resources, provided the availability of partners able to perform NGS analysis and computational analysis (Fig. 2E). As REMBRANDT uses an isothermal RNA reverse transcription and amplification reaction, it does not require PCR amplification. Moreover, since pairing of the template and primer during the amplification step relies on the activity of recombinases UvsX and UvsY, it is minimally affected by different *T*_*m*_ or *T*_*a*_ of the primers. Importantly, we find that this approach is effective on raw, unextracted patient samples, enabling REMBRANDT to bypass RNA extraction, while still producing values that are highly comparable to extracted patient samples. Current PCR-based testing requires RNA purification as raw samples show decreased sensitivity and significantly lower Ct values for SARS-CoV-2 amplicons. Importantly, REMBRANDT and LAMP-Seq pipelines can both contribute to COVID-19 mass screening strategies and ease some of the significant bottlenecks that are present in supply chain of RT-PCR based assays. The REMBRANDT pipeline also offers flexibility and can be readily adapted to detect other viral genes and/or species by switching the amplification regions of the primers. This is growing in importance: as new SARS-CoV-2 variants are identified; the REMBRANDT amplicon can be easily shifted to capture new regions required for variant testing. Based on our laboratory testing on raw and extracted patient samples, our results show that the REMBRANDT approach can accurately amplify and identify SARS-CoV-2 positive patients.

## Methods

### In vitro RNA transcription

To generate positive control RNAs, T7-flanked forward primers and standard reverse primers (Supplementary Table 1) were used in a PCR reaction to amplify a region of each target gene with Q5 polymerase. Templates used in this work were COVID-19-N (10006625, IDT) and RNase P (10006626, IDT). Gel purified PCR products (200 ng) were purified and used in T7 (Roche 10881775001) in vitro transcription reaction. DNase was then added for 15 min, before the RNA was precipitated using standard ethanol RNA purification method (8).

## Oligo plate preparation

Forward barcoding primers (plate-specific barcode) (Supplementary Table 1, N1 F) and barcoding primers (well-specific barcode) (Supplementary Table 1, N1 R) were dissolved at a 10 µM concentration in RNase-free water. 1 µl of each well-specific reverse primer was added to each well of a 96-well plate followed by the addition of 1 µl of a plate-specific forward barcoding primer.

## REMBRANDT steps

Prepare Isothermal Amplification Buffer 2X (IAB2X) (Table 4). Keep the 2X IAB2X mix on ice. Prepare the UvsX/UvsY mix by combining 10 ml of recombinant UvsX (5 mg/ml) and 10 ml of recombinant UvsY (2 mg/ml). Add 2 µl of the resulting mix to each well of the 96 well plates. We recommend gentle trituration 2–3 times of the UvsX/UvsY mix with the primers. This premixed plate should be kept on ice for all subsequent additions. Add 1 µl of template RNA to each well of the 96-well plates. Prepare the Enzyme Mix (Table 5). Keep the mix on ice. Proceed immediately to step. Dispense 14 µl of Enzyme Mix into the all wells of the of the 96-well plates. Keep on ice. Add 1 µl/well of 280 mM MgOAc to start the reaction. Place all the 96-well plates at 38^o^ C for 2 h. Collect the REMBRANDT products from all the 96-well plates into a single container (each 96-well plate will yield 1.92 ml). Add 5 volumes (9.6 ml) of DNA Binding Buffer. Mix well and load onto a Zymo-Spin VI column. Place the column(s) on a vacuum manifold, turn on the vacuum source and let the sample clear from the column (*repeat this step up to 5 times*). Add 5 ml of DNA wash Buffer. Repeat the wash step. Leave the vacuum source on for additional 5 min to remove all the wash buffer. Transfer the column into a 50 ml conical tube. Add 2 ml of water to the column and wait 1 min. Centrifuge at 3000×*g* for 3 min and collect the eluted barcoded DNA. If performing multiple plates/columns, combine the eluted barcoded DNA and label the combination as “Batch 1”. Subsequent Batches are individually used for library preparation and should not be combined until after ligation (Library construction) with batch specific adapters.

**Table 4:** Isothermal Amplification Buffer 2X (IAB2X).

Reagent	Stock concentration	Final concentration	Required volume 96 samples^#^
Tris Acetate pH 7.8	1 M	80 mM	92.8 µl
K-acetate	5 M	200 mM	46.4 µl
DTT	1 M	10 mM	11.6 µl
ATP	100 mM	5 mM	58 µl
dNTPs	10 mM	480 µM	55.68 µl
PEG-3500	44%	11%	290 µl
Trehalose	40%	10%	290 µl
Phosphocreatine	0.5 M	50 mM	116 µl
Creatine Kinase	2 µg/µl	200 ng/µl	116 µl
Acetylated BSA	10 mg/ml	200 µg/ml	23.2 µl
H2O	/	/	60.32 µl
Final volume			1.16 ml

**Table 5:** Enzyme mix.

Reagent	Stock concentration	Final Concentration	Required volume 96 samples#
T4 Gene 32 Protein	10 mg/ml	0.25 mg/ml	58 µl
Bsu DNA Polymerase	5000 U/ml	0.25 U/µl	116 µl
RevertAid Reverse Transcriptase	200 U/µl	10 U/µl	116 µl
SUPERase RNase Inhibitor	20 U/µl	1 U/µl	116 µl
IAB2X	2X	1X	1.16 ml
Water^##^			58 µl
Total volume			1.624 ml

## Library construction

Before library construction, a fraction of each purified pool of barcoded products should be examined using the Bioanalyzer to determine concentration and product size. The respective size of the COVID-19 product is 124 bp. Using the Illumina library construction kit (e7490), start at protocol 1.6 and conduct End repair on each pool of DNA fragments. Proceed to Adaptor Ligation Step. Proceed to Purification of Ligation of Reaction step. This is critical to remove any free adaptor that will add noise to the library construction. PCR Enrichment of Ligation Reaction. We recommend using 8 cycles to minimize over-amplification. Purification of PCR reaction. The overall library quality should be checked using a Bioanalyzer to confirm the size of the DNA fragments. Libraries that contain detectable levels of Primer-Primer annealing events should be repeated. Determine the absolute DNA concentration of each library using Qubit.

## Sequencing

The libraries are then loaded onto the Illumina sequencing platform and run as per manufacturer’s instructions. Once complete, the dataset is downloaded onto a suitable storage server for analysis.

## Computational methods

The REMBRANDT pipeline available on Github (https://github.com/MilesLab/Rembrandt_pipeline/) is coded mainly in the R programming language and consists of 2 main steps. The first step consists of aligning the reads to the N1 SARS COV2 and creating a ‘fastq’ file of those reads mapping to N1 SARS COV2. The second step involves detecting forward and reverse primer barcodes on those N1 SARS COV2 mapped reads and identifying reads that contain overlapping primer pairs.

The runalign.R script performs the alignment to N1 SARS COV2 using the align() function from the Rsubread Bioconductor package^20^. The N1 SARS COV2 sequence is available as a ‘fasta’ file in the data/ folder of the Github repository and buildindex() function can be utilized to prepare the index used to map reads to N1 SARS COV2. The mapped reads are then converted to a ‘fastq’ file using samtools^21,22^ and bedtools^23,24^. In addition, we also prepare a script that performs the conversion of mapped reads to ‘fastq’ using only the R programming language; however, this runs much slower.

The generate_overlap_matrix.R script detects forward and reverse primer barcodes and determines overlapping primer pairs. In the data/ folder, we provide files containing the list of forward and reverse primer pairs. Sequences from the N1 SARS COV2 ‘fastq’ files are imported using the FastqStreamer() and yield() functions from the ShortRead Bioconductor package^25^. A reverse complement of each of the reads is also determined. A string search is used to identify forward and reverse primer sequences in each of the reads and their reverse complement. An overlap matrix contains counts of primer-primer pairs present in a read and its reverse complement.

The Github repository contains detailed instructions and a test data set on how to set up and run the REMBRANDT pipeline (https://github.com/MilesLab/Rembrandt_pipeline/).

## PCR testing on patient samples

To determine positive and negative patient specimens for this study, we utilized the SARS-CoV-2 (2019-nCoV) CDC qPCR Probe Assay kits (Cat. # 10006770) manufactured by Integrated DNA Technologies, Inc. (Coralville, IA) and consistent with the most recent revision of the Emergency Use Authorization (EUA) issued to the CDC on December 1, 2020. This assay targets two nucleocapsid (N) gene regions with an additional primer/probe set to detect the RNase P gene (RP) in specimens. A cycle threshold (Ct) value of less than 40 for both N gene targets was considered positive for SARS-CoV-2 infection.

## Human samples

The US Air Force collected all COVID-19 patient samples as informed consent clinical specimens under protocols approved by the US Air Force. The Air Force Research Laboratory Institutional Review Board (Study number FWR20190037N) determined this study to be non-human subject research as part of a public health response activity and the study was conducted between March and June 2020.

## Supplementary Information

Supplementary Information.
